# Safety Evaluation of Crocin (a constituent of saffron) Tablets in Healthy Volunteers

**Published:** 2013-01

**Authors:** Amir Houshang Mohamadpour, Zahara Ayati, Mohammad–Reza Parizadeh, Omid Rajbai, Hossein Hosseinzadeh

**Affiliations:** 1 Pharmaceutical Research Center, Department of Pharmacodynamy and Toxicology, School of Pharmacy, Mashhad University of Medical Sciences, Mashhad, Iran; 2 Department of Pharmacodynamy and Toxicology, School of Pharmacy, Mashhad University of Medical Sciences, Mashhad, Iran

**Keywords:** Clinical trial, Crocin, Crocus sativus, Human volunteers, Safety assessment, Saffron

## Abstract

***Objective(s): ***Crocin is the chemical ingredient primarily responsible for the color of saffron. It has different pharmacological effects such as antioxidant, anticancer and memory improving activities. Crocin tablets were evaluated for short-term safety and tolerability in healthy adult volunteers.

***Materials and Methods: ***The study was a randomized, double-blind, placebo-controlled design consisting of one month treatment of crocin tablets. Volunteers who fulfilled inclusion and exclusion criteria were randomized into 2 groups of 22 each (males and females) and received 20 mg crocin tablets or placebo. General measures of health were recorded during the study such as hematological, biochemical, hormonal and urinary parameters in pre and post-treatment periods.

***Results: ***No major adverse events were reported during the trial. Crocin tablets did not change the above parameters except that it decreased amylase, mixed white blood cells and PTT in healthy volunteers after one month.

***Conclusion: ***This clinical safety evaluation showed a relatively safe and normal profile for crocin in healthy volunteers at the given doses within the trial period.

## Introduction

Saffron (*Crocus sativus*, L.) has been used traditionally as a coloring or flavoring agent, as well as a herbal remedy. It contains four major bioactive constituents which are crocin (color), crocetin (color), picrocrocin (taste) and safranal (aroma) (1). 

Crocin is a carotenoid of constituent of saffron has also shown various pharmacological activities such as antioxidant (2), anticancer (3-4), memory improvement (5-6), antidepressant (7), cerebral (8), kidney (9), heart (10), skeletal muscle (11)) anti-ischemia, hypotensive (12), aphrodisiac (13), genoprotective (14) and antidote activities (15-17). Crocin also inhibit morphine withdrawal syndrome (18) and morphine-induced reinstatement of place preference in mice **(**19**)**.

The acute and sub-acute toxicity of crocin was evaluated in mice and rats. At pharmacological doses, crocin did not exhibit marked damages to all major organs of the body. With high doses (3 g/kg, IP or orally) after 24 and 48 hr no mortality was seen by crocin in mice (20). According to Loomis and Hayes (21) classification, chemical substances with LD_50_ values within the range of 1–5 g/kg are considered as practically low-toxic.

Since, there is insufficient data on toxicological evaluation of crocin in human for giving safety assurance in developing this constituent of saffron as a medicine, in this study crocin was evaluated for short-term safety and tolerability in healthy adult volunteers.

## Materials and Methods

***Study design***

This study was a double-blind, randomized, placebo-controlled study consisting of one month treatment. The study was approved by the Ethics Committee of MUMS (Mashhad University of Medical Sciences). All subjects were asked to submit a signed consent form before participating in the study. This study was carried out at Imam Reza Hospital in association with Pharmaceutical Research Center, Department of Pharmacology and Toxicology of Mashhad University of Medical Science from May 2009 to January 2010. 

Forty two volunteers who fulfilled the following inclusion and exclusion criteria were enrolled in the study. All participants provided written informed consent prior to the start of the study. This form consisted of demographic data, previous medical history, drug history and familial history. The volunteers were randomized into 2 groups of 22 each who received 20 mg crocin tablets or placebo for 1 month. Before and after treatment clinical examination and laboratory tests (hematology, blood biochemistry and hormone and urine analysis) were carried out. During clinical trial the volunteers were evaluated for adverse drug reactions in both groups. 

The dose of crocin (20 mg) was selected based on acute and sub-acute toxicity of crocin in mice and rats (20) as well as a recent study on the oral administration of crocetin (15 mg, 8 days) that attenuated physical fatigue in men compared with placebo (22).

***Inclusion & exclusion criteria***

Healthy adult volunteers who were willing to give voluntary written informed consent were included in this study. The volunteers were proven to be healthy through clinical examination by the physician along with hematological and biochemical investigations. 

Volunteers were excluded from study if any of the following criteria occurred at the time of study: 1- History of allergy to saffron 2- History of blood disease, for example: Iron deficiently, anemia, hemophilia, 3- History of cardiovascular disease including vascular or congestive cardiac disease, hypertension, orthostatic hypotension, 4- History of renal disorder or electrolytes disorder. 5- History of endocrine disease such as thyroid disorder, diabetic mellitus, dyslipidemia, 6- History of gynecologic or any menstrual disturbance, 7- Pregnant or lactating women 8- Addiction to smoking or any other substance. 9- Volunteers taking any drugs.

***Blood sampling and laboratory investigations***

Five ml blood sample was taken from each volunteer. 1.5 ml was used for hematologic tests and blood sample of 3.5 ml were collected in sterile tubes, centrifuged at 2500 rpm for 10 min, and immediately cooled to −20°C for 30 min, and then stored at −70°C until the time of analysis.

The following laboratory tests were determined in both groups before and after treatment:

Hematologic tests consisted of complete blood count with differentiation (CBC diff), serum iron plasma lipids (total cholesterol (HDL-c, LDL-c), triglyceride), liver function tests (serum glutamic oxaloacetic transaminase (SGOT), serum glutamic pyruvic transaminase (SGPT), serum bilirubin, total and direct, albumin), kidney function tests (urea, creatinine, urine analysis) electrolytes (calcium, phosphorus, sodium, potassium), coagulation tests (PTT, PT, INR), endocrine evaluation tests (fasting blood glucose, T4, TSH, cortisol, testestron), enzymes (alkaline phosphatase, lactate dehydrogenase, creatinine phosphokinase, amylase, lipase), inflammation biomarkers (hs-CRP, ESR ) and uric acid.

***Crocin extract and purification***

Stigmas of *C. sativus *L. from Novin Saffron (collected from Ghaen, Khorasan province, Northeast of Iran) was obtained and analyzed in accordance with the ISO/TS 3632-2. Crocin was extracted and purified as defined by Hadizadeh and colleagues) 23).

***Statistical analysis***

Data (the diference between after-before treatment) are expressed as mean±SD. Statistical analysis was done using two independent sample test with SPSS 11.5 software. The *P*-values less than 0.05 were considered to be statistically significant. 

## Results

***Adverse effects***

There were two drop outs during study period in crocin group. No major adverse events were reported during the trial. However, three volunteers showed adverse effects in crocin group. One volunteer claimed that had a burning symptom on his kidney. He had a history of kidney stone. Another volunteer demonstrated somnolence and urinary frequency and had occasional a burning sensation in his body for twenty min 30 min after receiving crocin tablet. Also, one woman volunteer had **heavy** and prolonged menstrual **bleeding** during her menstrual period. She had an early menstrual period. These symptoms were omitted without any need for discontinuation of treatment.

***Characteristics of the study population***

Demographic data including age, sex, weight, and body mass index were reported in Table 1. All volunteers have normal range of laboratory tests before entering the study.

***Effect of crocin on blood cells and inflammatory biomarkers in healthy adult volunteers***

The difference in RBC count, WBC, and platelet and their indices as well as inflammatory biomarkers after 1 month treatment were compared between 2 groups. There was a significant reduction of MXD count in crocin group in comparison with control group but there were no significant differences between 2 groups with respect to other blood cells (Table 2).

The difference of PT and aPTT after 1 month treatment were compared between 2 groups. There was a significant reduction of aPTT in crocin group in comparison with control group but there were no significant differences between 2 groups with respect to PT (Table 2).

***Effect of crocin on some of important enzymes in healthy adult volunteers***

The difference of enzymes activity (alkaline phosphatase, lactate dehydrogenase, Creatinine phosphokinase, amylase, lipase) after 1 month treatment were compared between 2 groups. There was a significant reduction of amylase activity in crocin group in comparison with control group but there were no significant differences between 2 groups with respect to other enzymes activity (Table 3).

**Table 1:** Demographic data of volunteers

Total population number	42
Percentage of male volunteers	72.7%
Percentage of female volunteers	27.3%
Age (Years)	31.1 ± 13
Weight (Kg)	66.59 ± 15
BMI (Kg/m^2^)	24.9 ± 7.1

**Table 2:** Effect of crocin tablets on hematological indices in healthy adult volunteers. Crocin tablets (20 mg/day) were administrated for one month. Values presented as the mean±SEM; n=20-22 in each group. Two Independent Sample T test. Δ= Post treatment– Pre treatment

	Placebo group	Crocin group	*P*-value
Δ WBC:**White blood cells** (x1000/microLiter)	-0.14 ± 0.28	-0.12 ± 0.18	0.947
Δ Lymph%	-1.8 ± 1.86	1.43 ± 1.48	0.187
Δ MXD%	2.55 ±0.91	-0.347 ± 1.02	0.042
Δ NEUT: **neutrophils** %	1.5± 2.9	-1.08 ± 1.65	0.465
Δ Lymph (x1000/µl)	-0.11 ± 0.15	0.026 ± 0.07	0.442
Δ MXD^1^ (x1000/µl)	0.18 ± 0.07	-0.031 ± 0.067	0.037
Δ NEUT:**neutrophils** (x1000/µl)	0.009 ± 0.29	-0.11 ± 0.32	0.746
Δ PLT :platelet (x1000/µl)	21.7 ± 14.8	11.05 ±6.59	0.523
Δ PDW^2^ (fl)	1.15 ±0.72	-0.21 ± 0.17	0.128
Δ MPV^3^ (fl)	0.60 ± 0.52	0.11 ± 0.2	0.24
Δ PT:**prothrombin** **time** (sec 100%)	-0.76± 0.16	0.8 ± 0.44	0.924
Δ PTT:**partial** **thromboplastin** **time** (sec)	2.66 ± 1.03	-0.42 ± 0.85	0.029
Δ RBC: Red Blood Cell(x1000000/µl)	-0.09 ± 0.06	-0.17 ±0.108	0.477
Δ Hgb:hemoglobin (g/dl)	-0.15 ± 0.181	-0.09 ±0.18	0.811
Δ HCT:hematocrit (%)	-0.51 ± 0.49	0.59 ±0.55	0.924
Δ MCV: **mean cell volume** (fl)	0.62 ± 0.21	0.52 ± 0.32	0.789
Δ MCH: **Mean** Corpuscular **Hemoglobin** (pg)	-0.26 ± 0.8	-0.49 ± 0.45	0.105
Δ MCHC:**Mean Corpuscular Hemoglobin Concentration** (g/dl)	0.043 ± 0.13	0.24 ± 0.106	0.256
Δ RDW-SD^4^ (fl)	2.99 ± 2.02	0.089 ± 0.39	0.189
Δ RDW-CV^5^ (%)	0.68 0.605	-0.068 ±0.14	0.259
Δ Serum Iron (µg/dl)	-29.7± 11.54	-15.26 ±8.3	0.319
Δ hs-CRP :high sensitivity C Reactive Protein (mg/dl)	-0.05± 0.49	0.25 ± 0.609	0.693
Δ Serum Iron (µg/dl)	-29.7± 11.54	-15.26 ±8.3	0.319
Δ ESR :erythrocyte sedimentation rate (mm/h)	-0.42 ± 1.06	-1.6 ± 0.96	0.404

**1- MXD% - relative (%) content of the mixture (the norm is 5 - 10%),** **monocytes** **,** **basophils** **, and** **eosinophils**

**2- PDW** **- the relative width of the distribution of platelets in volume index of** **the heterogeneity of** **platelets**

**3- MPV** **(mean platelet volume) - the average volume** **of platelets**

**4- RDW-SD - the relative distribution width** **of red blood cells** **by volume,** **standard deviation**

**5- RDW-CV - the** **relative distribution width** **of red blood cells** **by volume,** **coefficient of variation**

**Table 3:** Effect of crocin tablets on some important enzymes in healthy adult volunteers. Crocin tablets (20 mg/day) were administrated for one month. Values presented as the mean±SEM; n=20-22 in each group. Two Independent Sample T test. Δ= Post treatment– Pre treatment

	Placebo group	Crocin group	*P*-value
Δ ALP: alkaline phosphatase (U/L)	5.95 ± 9.45	-12.5±8.79	0.162
Δ LDH:lactic dehydrogenase (U/L)	-11.76 ± 17.85	-50.35±35.96	0.335
Δ CPK: Creatine Phosphokinase (UI/I)	-32.23 ± 43.92	-15.2±8.3	0.206
Δ Amylase (UI/I)	0.606 ± 2.6	-8.5±2.4	0.015
Δ Lipase (IU/l)	26.6 ±21.4	56.35±85.8	0.753

**Table 4:** Effect of crocin tablets on biochemistry function of kidney and serum electrolytes in healthy adult volunteers. Crocin tablets (20 mg/day) were administrated for one month. Values presented as the mean ± SEM; n=20-22 in each group. Two Independent Sample T test. Δ= Post treatment– Pre treatment

	Placebo group	Crocin group	*P*-value
Δ Urea (mg/dl)	-1.27 ± 1.51	1.3± 1.52	0.239
Δ Creatinine (mg/dl)	-0.13 ± 0.02	0.1 ± 0.03	0.354
Δ Uricacid (mg/dl)	-0.36 ± 0.13	0.25 ± 0.15	0.595
Δ Calcium (mg/dl)	-0.69 ± 0.23	0.61 ± 0.189	0.812
Δ Phosphorus (mg/dl)	-2.19 ± 2.2	-0.14± 0.07	0.381

***Effect of crocin on kidney function tests and serum electrolytes in healthy adult volunteers***

The difference of kidney function tests (BUN, serum Creatinine, urine analysis) and some important serum electrolytes (calcium and phosphorus) after 1 month treatment were compared between 2 groups. There were no significant differences between 2 groups with respect to these parameters (Table 4).

***Effect of crocin on liver function tests in healthy adult volunteers***

The difference of liver function tests (SGOT, SGPT, serum bilirubin-total and direct, Albumin) after 1 month treatment was compared between 2 groups. There were no significant differences between 2 groups with respect to these parameters (Table 5).

***Effect of crocin on some of endocrine laboratory tests in healthy adult volunteers***

The difference of some of endocrine laboratory tests (FBS, T4, TSH, cortisol, and testestron) after 1 mo treatment was compared between 2 groups. There were no significant differences between 2 groups with respect to these parameters (Table 6).

***Effect of crocin on serum lipid profile in healthy adult volunteers***

The difference of serum lipid profile (total cholesterol (HDL-c, LDL-c), triglyceride) after 1 month treatment was compared between 2 groups. There were no significant differences between 2 groups with respect to these parameters (Table 7).

**Table 5:** Effect of crocin tablets on liver function in healthy adult volunteers. Crocin tablets (20 mg/day) were administrated for one month. Values presented as the mean ± SEM; n=20-22 in each group. Two Independent Sample T test. Δ= Post treatment– Pre treatment

	Placebo group	Crocin group	*P*-value
Δ SGOT: Serum Glutamic Oxaloacetic Transaminase (U/l)	-1.68 ± 1.28	5.94 ± 2.29	0.101
Δ SGPT: Serum Glutamic Pyruvic Transaminase (U/l)	-1.5 ± 0.61	-7.89 ±4.07	0.103
Δ Total Billirubin (mg/dl)	0.13 ± 0.04	0.14 ± 0.06	0.956
Δ Direct Billirubn (mg/dl)	0.0043 ± 0.01	0.02 ± 0.055	0.327
Δ Albumin (g/dl)	-0.1105 ± 0.08	-0.123 ± 0.088	0.914

**Table 6:** Effect of crocin tablets on some of endocrine laboratory levels in healthy adult volunteers. Crocin tablets (20 mg/day) were administrated for one month. Values presented as the mean ± SEM; n=20-22 in each group. Two Independent Sample T test. Δ= Post treatment– Pre treatment

	Placebo group	Crocin group	*P*-value
Δ FBS : Fasting Blood Sugar (mg/dl)	-3.6 ± 1.16	0.3 ± 2.52	0.152
Δ T4: Thyroxine (µg/dl)	0.045 ± 0.28	0.63 ± 0.207	0.109
Δ TSH: Thyroid-stimulating hormone ( (µIU/ml)	0.35 ± 0.17	0.2 ±0.24	0.595
Δ Cortisol (µg/dl))	-1.62± 1.27	-4.96 ±1.59	0.108
Δ Testestron (mg/dl)	24.1 ± 16.44	4.5± 45.07	0.675

**Table 7:** Effect of crocin tablets on lipid profiles in healthy adult volunteers. Crocin tablets (20 mg/day) were administrated for one month. Values presented as the mean ± SEM; n=20-22 in each group. Two Independent Sample T test. Δ= Post treatment– Pre treatment

	Placebo group	Crocin group	*P*-value
Δ Cholestrol (mg/dl)	-9.5 ± 4.85	-17.5 ± 6.3	0.314
Δ TG:Triglyceride. (mg/dl)	3.04± 9.66	0.1± 12.7	0.853
Δ HDL-C:high-density lipoprotein cholesterol (mg/dl)	-2.36 ± 1.5	-1.2 ± 1.48	0.603
Δ LDL-C :low-density lipoprotein cholesterol (mg/dl)	-4.04 ± 2.59	-8.95 ± 2.87	0.212

## Discussion

The result of present study showed that crocin tablet (20 mg/day, one month) could decrease the amylase, PTT and MXD in the healthy adult volunteers.

Few studies have evaluated safety of saffron or its constituents in human. In one study, saffron (*Crocus sativus*) stigma tablets were evaluated for short-term safety and tolerability in healthy adult volunteers. In this study, volunteers had 200 mg and 400 mg saffron tablets for seven days. Saffron tablets changed some hematological and biochemical parameters (24). There are also some clinical trials about saffron for assessment of its activity such as antidepressant (25), improving male erectile dysfunction (26) and anti-Alzheimer (27). However, it seems there is no safety evaluation about the constituents of saffron such as crocin, crocetin or safranal. In one double-blind, placebo-controlled, 3-way crossover study, the oral administration of crocetin (15 mg, 8 days) attenuated physical fatigue in men compared with placebo (22).

In this study, crocin decreased **MXD**% and level (**monocyte**, eosinophil and basophil) without any effects on total white blood cells count as well as neutrophil and lymphocyte. Kianbakht and Ghazavi (28) showed that saffron decreased the percentage of basophils but increased the percentage of monocytes compared with placebo after 3 weeks. However, these parameters returned to the baseline levels after 6 weeks. In animal study, the aqueous extract of saffron stigma did not change WBC after 14 days treatment in rats (29). In a recent study, the hematological parameters such as WBC, RBC, Hg and Ht did not alter by crocin (15-180 mg/kg) in rats (20). 

The administration of 20 mg/d crocin ay for one month decreased activated partial thromboplastine time (aPTT). In one placebo‐controlled clinical study was found the administration of 200 and 400 mg saffron tablets to healthy adult volunteers (n = 10 in each of the three groups) for 7 days significantly reduced the platelet count (only a dose of 200 mg/day). Saffron tablet at a dose of 200 mg also decreased platelets, INR and bleeding time. However, crocin prolonged blood coagulation time of mice and markedly inhibited, dose-dependently, thrombin- and ADP-induced blood platelet aggregation of rabbits *in vivo* (30). Crocetin inhibited platelet aggregation induced by ADP and collagen, without effect on platelet aggregation induced by arachidonic acid (31). Thus, further study in a larger sample size and longer time period is needed to clarify this discrepancy.

A reduction in amylase level was considered in crocin group. *Gardenia jasminoides* whose main components contain geniposide and crocin reduced pancreatic edema, neutrophil infiltration, serum amylase and lipase levels, serum cytokine levels, and mRNA expression by cerulein-induced acute pancreatitis (32).

Japanese researchers showed that crocins (orally) were hydrolyzed to crocetin before or during intestinal absorption (33). In another study, Xi *et al*, (34) showed that the orally administrated crocin (single or repeated doses 6 days) is not absorbed via the intestinal tract of rats and is mainly excreated through the colon. The low concentrations of crocetin (by hydrolysis of crocin) were also detected in plasma (34). In mice, the oral administration of crocin up to 3 g/kg did not induce any mortality after 48 hr (20). Thus it seems that most dosage of crocin excretes via colon and a small amout converts to crocetin in animals. However, pharmacokinetic effect of crocin including its oral absorbtion in human needs to be studied. 

Crcoin selectively inhibited the activity of pancreatic lipase in the intestine and reduced serum triglyceride, total cholesterol, low density lipoprotein (LDL) cholesterol and very low density lipoprotein (VLDL) cholesterol level in the daily oral dose range of 25 to 100 mg/kg in rats. Thus, the oral administration of crocin will be suitable as a hypolipidemic medicine (35).

This clinical safety evaluation showed a relatively safe and normal profile for crocin in healthy volunteers at the given doses within the trial period (20 mg/day, one month). However, further investigations need to be done with higher doses, longer period of study and larger number of participants.
